# Temporal and Spatial Heterogeneity of Soil Erosion and a Quantitative Analysis of its Determinants in the Three Gorges Reservoir Area, China

**DOI:** 10.3390/ijerph17228486

**Published:** 2020-11-16

**Authors:** Lin Chu, Tiancheng Sun, Tianwei Wang, Zhaoxia Li, Chongfa Cai

**Affiliations:** College of Resources and Environment, Huazhong Agricultural University, Wuhan 430070, China; chulin@mail.hzau.edu.cn (L.C.); tiancheng_hzau@sina.com (T.S.); wangtianwei@webmail.hzau.edu.cn (T.W.); cfcai@mail.hzau.edu.cn (C.C.)

**Keywords:** soil erosion, CSLE, spatial heterogeneity, geographical detector, determinants, TGRA

## Abstract

As the most typical ecologically fragile area in South China, the Three Gorges Reservoir Area (TGRA) suffers from water and soil loss, which has threatened the local ecological environment. Understanding the spatial heterogeneity of soil erosion and exploring its determinants are of great significance in preventing soil erosion and maintaining ecological sustainability in the TGRA. This study investigates the spatial heterogeneity of soil erosion and quantitatively identifies the determinants in the TGRA based on the Chinese Soil Loss Equation (CSLE) and geographical detector method. This study concluded that the soil erosion status generally improved from 1990 to 2015, showing an increasing trend from 1990 to 2000 and a decreasing trend from 2000 to 2010. Slope, land use, and vegetation coverage were the dominant individual factors affecting soil erosion in the TGRA. For the interaction factor, the combinations of land-use type and slope and vegetation coverage and slope were the key determinants, explaining 68.7% and 63.1% of the spatial heterogeneity of soil erosion in the TGRA from 1990 to 2015, respectively. Moderate and higher levels of soil erosion occurred in areas where the slope was greater than 25°. Among the land-use types, dry land and bare land were prone to soil erosion. These findings reveal that land-use type and vegetation coverage should be considered for the effective prevention of soil erosion, and cultivation on sloped farmland should be prohibited, especially on slopes higher than 25° in the TGRA.

## 1. Introduction

Soil erosion is a major global environmental issues that limits socioeconomic and environmental sustainability [1]. Soil erosion is an important form of non-point source (NPS) pollution and a primary transport mechanism that introduces a large amount of sediment and nutrients into various water bodies, causing water environment deterioration and thus endangering public health [2,3,4]. Many challenges are associated with soil erosion, such as land resource destruction and frequent calamities [5]. Affected by natural and anthropogenic aspects, the occurrence and development of soil erosion involve complex processes that have significant spatial heterogeneity [6,7]. Therefore, investigating the spatial heterogeneity of soil erosion and quantifying its determinants are, therefore, of great significance for mitigating soil erosion, maintaining the ecological balance, and achieving regional sustainable socioeconomic development.

The accurate estimation of soil erosion is a prerequisite for investigating its determinants. To quantitatively evaluate and predict soil erosion, different mathematical models have been established, including physical process models and empirical statistical models. Physical process models such as the Water Erosion Prediction Project (WEPP) [8], Limburg Soil Erosion Model (LISEM) [9], European Soil Erosion Model (EUROSEM) [10], and Griffith University Erosion System Template (GUEST) [11] have complex structures and restrict the input parameters. Compared with physical process models, empirical statistical models are widely used worldwide and have the advantages of a simple structure, few input parameters, easy acquisition of required data, and strong applicability [12]. The Universal Soil Loss Equation (USLE) [13] and Revised Universal Soil Erosion Equation (RUSLE) [14] are two typical representatives of empirical statistical models. Several studies have demonstrated the ability of the USLE and RUSLE to assess and predict soil erosion globally [15,16]. However, considerable errors occur when applying the USLE and RUSLE to steeper areas with slopes greater than 18° [17,18]. To improve the simulation accuracy in regions with steep slopes, the Chinese Soil Loss Equation (CSLE) [19] was built by modifying the USLE/RUSLE, and it is suitable for estimating soil loss in areas with slopes less than 55°. The CSLE is now widely applied for soil erosion assessment in China, especially in mountainous areas [20,21,22].

Both natural and anthropogenic components are included in the CSLE model. The occurrence and development of soil erosion processes are largely determined by natural conditions. Natural factors such as climate [23], soil properties [24], topography [18,25,26], geology [27], and vegetation [28] exert significant influences on soil erosion. Among them, precipitation is a vital factor and directly affects soil erosion by forming soil splashes, generating surface runoff and scouring soil [29,30]. Related studies have shown a significant positive correlation between the total soil erosion amount and precipitation intensity and between the total amount of soil erosion and precipitation duration [31,32]. Zhao et al. have found that, in southern China, the precipitation intensity of 30 mm/h over a duration of 1 h was not large enough to trigger soil erosion on slight (5°), moderate (15°) and steep (25°) slopes, and they showed that soil erosion occurred when the precipitation intensity exceeded 60 mm·h^−1^, [33]. A precipitation intensity from 70 mm·h^−1^ to 95 mm·h^−1^ over a duration of 1 h is very common in sub-humid climate regions of China that are dominated by monsoon climate conditions [34]. The soil physical properties—such as the soil structure, the particle composition, and the thickness of the soil layer [35,36]—affect the soil erosion resistance and corrosion resistance and determine the soil erosion degree [28]. Zhang et al. have found that soil erosion significantly varies when the sand layer exceeds 5 cm under the controlled conditions of precipitation intensity (90 mm·h^−1^) and slope gradient (27°) on the Loess Plateau of China [37]. As another direct driving force that affects surface runoff, the topography (e.g., the slope gradient, the slope length and the slope aspect), changes the processes of physical forces and consequently determines the latency capability for soil erosion [38,39,40]. The soil erosion amount increases with the slope gradient, especially when the slope exceeds 10° [41,42,43]. As a positive and effective factors for soil erosion control, vegetation greatly impacts hydrological processes by intercepting rainfall, regulating surface runoff, altering the soil erosion process by consolidating soil, and improving soil properties [44,45].

Human factors have complex space–time effects on soil erosion that cannot be ignored. Human factors alter the soil erosion degree, spatial pattern, and evolution process to some extent. Human activities will have both positive and negative influences, leading to a more complicated soil erosion development process. The implementation of positive anthropogenic measures—such as engineering measures, rotation measures, and management factors—can effectively control the occurrence and development of soil erosion [46,47]. Irrational land use is catalysts for accelerating soil erosion, and examples include deforestation, reclamation of grassland, and down-slope cultivation [48]. In addition to adverse natural conditions and irrational human activities, their interactions have a great impact on soil erosion development.

Previous studies have focused on the impacts of certain factors on soil erosion by using traditional approaches, such as correlation analysis, regression statistical methods, controlled trial, variable-controlled approaches, etc. [28,36,39,49]. These approaches have contributed significantly to revealing the impacts of certain factors on soil erosion at the field scale. However, few previous studies have performed quantitative attributions of the multi-factor influences and their interaction. In addition, most studies have been conducted at the field scale, with few at the regional scale. As a new statistical approach, the geographical detector reveals the driving force behind the spatial stratification heterogeneity of geographic phenomena, and this method is currently applied in environmental sciences [50].

The Three Gorges Dam (TGD), the most controversial water conservancy project in China, has a far-reaching influence on both the regional ecological environment and socioeconomic development [51]. The economic development of the Yangtze River basin grew rapidly after the impoundment of the Three Gorges Reservoir. Due to adverse natural conditions and irrational human activities, soil erosion has occurred in the Three Gorges Reservoir Area (TGRA), which has threatened the region’s residents and the Three Gorges Reservoir [52]. The TGRA is not only the most fragile ecological area in China but also a key zone for soil erosion control [53]. To better explain the factors that influence soil erosion, it is necessary to identify the soil erosion heterogeneity in the TGRA across temporal and spatial scales. The primary goal of this study, therefore, was to estimate the soil erosion amount using the CSLE model from 1990 to 2015 and to quantitatively distinguish its influencing factors using the geographical detector method. These findings can offer a scientific reference for soil erosion control and eco-environmental conservation in the TGRA and other similar ecologically sensitive areas.

## 2. Materials and Methods 

### 2.1. Study Area

**Figure 1:**
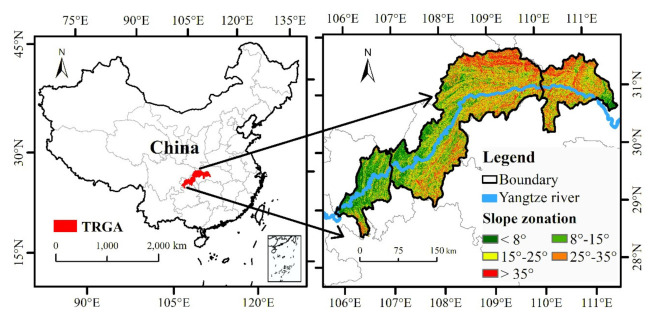
Geographical location of the study area.

The TGRA extends from 105.72° to 111.68° E longitude and 28.52° to 31.74° N latitude, and the TGRA has 21 counties (cities and districts) along the Yangtze River from Hubei Province and Chongqing Municipality, covering an area of 5.85 × 10^4^ km^2^ (Figure 1). According to differences in the natural conditions in the TGRA, the study region is divided into three parts by administrative boundaries: the head segment, middle segment and end segment of the TGRA. The overall terrain of the TGRA is very complex and shows characteristics of being low in the east and high in the west, with a maximum elevation of 2973 m. As the dominant terrain type, mountains account for approximately 74% of the total area. The study region enjoys moderate temperatures and a subtropical humid monsoon climate, and rainfall is unevenly distributed throughout the year. There are several soil types in the TGRA, mainly including purple soil, limy soil, yellow soil, and yellow brown soil.

### 2.2. Data Collection and Prepossessing

The land-use dataset is available from the Data Center for Resources and Environmental Sciences, Chinese Academy of Sciences (RESDC) website (https://www.resdc.cn), and the data have a spatial resolution of 30 m. Land-use data of the study area for the years 1990, 1995, 2000, 2005, 2010, and 2015 were selected. There are 6 primary classifications and 25 secondary classifications in the corresponding land use category system.

Two kinds of normalized difference vegetation index (NDVI) datasets were selected to calculate vegetation coverage. One is the NOAA Advanced Very High-Resolution Radiometer (NOAA AVHRR) product, and the other is the Moderate Resolution Imaging Spectroradiometer (MODIS) product. The AVHRR NDVI has a temporal resolution of 10 days and a spatial resolution of 8 km, covers the period from 1990 to 2005, and can be downloaded from the NASA website (ftp://daac.gsfc.nasa.gov/data/avhrr/global_8km/). The MODIS NDVI is derived from the MOD13Q1 product, has a temporal resolution of 16 days and a spatial resolution of 250 m, covers the period from 2000 to 2015, and is available from the official USGS website (https://lpdaac.usgs.gov/). A processing sequence was performed on the AVHRR and MODIS NDVI datasets, and this sequence included atmospheric correction, geometric correction and resampling. Due to large differences in the spatial and temporal resolutions, inevitable errors will be generated by using NDVI data from different sensors. The AVHRR NDVI from 1990 to 1995 was resampled to a spatial resolution of 250 m by establishing a linear regression equation between the MODIS NDVI and AVHRR NDVI. All NDVI data for a whole year were averaged as the annual NDVI. Six phases of NDVI data were obtained: 1990, 1995, 2000, 2005, 2010, and 2015. According to the field investigation, the vegetation coverage was calculated, which is described using Equation (1)

(1) $$vfc=\frac{\left(NDVI-NDVI_{soil}\right)}{\left(NDVI_{veg}-NDVI_{soil}\right)}$$

where vfc represents the vegetation coverage,$\;NDVI_{soil}$ refers to the NDVI value of soil without vegetation coverage or of bare soil, and $NDVI_{veg}$ is the NDVI value of the grid fully covered by vegetation.

The digital elevation model (DEM) dataset with a spatial resolution of 30 m is available from the Land Processes Distributed Active Archive Center (LP DAAC) of the United States Geological Survey (USGS) website (https://gdex.cr.usgs.gov/gdex/), and this dataset was derived from the ASTER Global Digital Elevation Model (ASTER GDEM) Version 002 product. Re-projection and mask processing were performed. Then, both the slope map and the aspect map were generated by using tools in ArcGIS 10.4 (ESRI, Redlands, California, USA).

The soil dataset with a spatial resolution of 1 km was provided by the Cold and Arid Regions Sciences Data Center at Lanzhou, China (http://westdc.westgis.ac.cn), and the dataset includes information on the soil physical properties and soil types.

Meteorological data between 1980 and 2015 from 126 weather stations are available from the China Meteorological Data Service Center (https://data.cma.cn/). Monthly and yearly precipitation data were selected to obtain R values in the CSLE model. The yearly average air temperature and precipitation data were selected for a quantitative analysis of the soil erosion determinants. Meteorological data were spatially interpolated using the kriging method to attain a spatial resolution of 30 m. The verification results agree with the accuracy demands, as determined by cross-validation.

Population data were obtained from the statistical yearbook at the county level. Population density was determined by dividing the population by the corresponding area of each county.

Night-time light data are highly correlated with socioeconomic status. As a socioeconomic indicator, night-time light data have been widely used to estimate gross domestic product (GDP) [54]. Different night-time light data were selected due to the long temporal span. The data included the Defense Meteorological Satellite Program (DMSP) Operational Linescan System (OLS) product and the Visible Infrared Imaging Radiometer Suite (VIIRS) Day/Night Band (DNB) product. Two kinds of night-time light data are available from the National Oceanic and Atmospheric Administration (NOAA) website (https://ngdc.noaa.gov/eog/download.html). Note that due to the limited launch time of the DMSP satellite, the night-time light data of 1992 were used instead of the 1990 data. The yearly data from 1992 to 2015 were chosen. After reprojection, masking, and resampling, night-time light images of six years were obtained and had a spatial resolution of 500 m.

### 2.3. Methods

#### 2.3.1. Chinese Soil Loss Equation (CSLE)

The CSLE model has been widely adopted to assess soil erosion in China, especially in mountainous areas [16,18]. A detailed description of the CSLE model is shown in Equation (2)

(2) A=R⋅K⋅L⋅S⋅B⋅E⋅T

where *A* is the annual soil erosion module ($t\cdot {\mathrm{hm}}^{-2}\cdot a^{-1}$), *R* represents the rainfall erosivity factor ($\mathrm{MJ}\cdot \mathrm{mm}\cdot {\mathrm{hm}}^{-2}\cdot h^{-1}\cdot a^{-1}$); *K* refers to the soil erodibility factor ($t\cdot h\cdot {\mathrm{MJ}}^{-1}\cdot mm^{-1}$); *L* and *S* are the slope length and slope steepness factor, respectively; *B* refers to the biological control factor, *E* represents the engineering-control factor, and *T* is the tillage practices factor. Note that the units of *L*, *S*, *B*, *E* and *T* are dimensionless and range from 0 to 1. Parameters in the CSLE model were determined as follows.

Based on the interpolation results of daily precipitation, the *R* factor was evaluated by using the calculation approach proposed by Wischmeier et al. [55].

The *K* factor was computed by using method proposed by Williams et al. [56], and this method requires soil organic carbon and soil particle composition data.

The *S* and *L* factors were estimated based on methods proposed by McCool et al. [26] and Liu et al. [39].

The *B* factor is largely determined by vegetation coverage and vegetation type. Cai et al. [15] quantitatively established the relationship between field-measured *B* values and vegetation coverage in the TGRA. Based on this relationship, the *B* value was estimated.

The *E* value was determined according to the land-use type, slope, and engineering-control measures. Combined with actual survey data and the statistical yearbook of the TGRA, the proportion of cultivated land by ridge terraces and stone terraces in the counties of the TGRA between 1990 and 2015 was calculated. According to the proportion, the *E* value of cultivated land from 1990 to 2015 was assigned.

The announcement of “the First Chinese Water Conservancy Survey on Soil and Water Conservation” was officially released in 2013, in which the *T* factor in China was investigated, and its value was determined based on long-term monitoring data for the TGRA. According to the agricultural statistical yearbook of the TGRA, the major crop types and planting areas at the county scale of the TGRA between 1990 and 2015 were collected. The proportion of cultivated land by major crops in the counties of the TGRA between 1990 and 2015 was calculated. Finally, the *T* value of cultivated land was modified and assigned according to the proportion.

The raster layers of the above seven factors (*R*, *K*, *L*, *S*, *B*, *E*, and *T*) are resampled to a spatial resolution of 30 m. According to the nationally standardized soil erosion grades in China, we divided the annual soil erosion modules into six grades: slight, minor, moderate, intense, very intense, and extreme (Table 1).

#### 2.3.2. Geographical Detector

The spatial heterogeneity of natural and socioeconomic processes and associated driving mechanisms can be easily identified and revealed by the geographical detector method. Compared with traditional mathematical statistical models, the geographical detector method has fewer assumptions and smaller data requirements [57]. Moreover, it can distinguish both qualitative and numerical data. This method consists of four modules: factor detector, interaction detector, risk detector, and ecological detector. To quantitatively investigate the determinants and interactions that affect the spatial heterogeneity of soil erosion in the TGRA, the factor detector module, and interaction detector module were used in this study.

In the factor detector module, the spatial stratified heterogeneity and determinant power can be assessed by the q-statistic, which is described by Equation (3)

(3) $$\begin{array}{c} q=1-\frac{\sum_{h=1}^{L}N_{h}\sigma_{h}^{2}}{N\sigma^{2}}=1-\frac{SSW}{SST}\\ SSW=\sum_{h=1}^{L}N_{h}\sigma_{h}^{2},\;SST=N\sigma^{2} \end{array}$$

where q represents the relevance between *Y* and *X*, ranging from 0 to 1. $N_{h}$ is the number of units in strata h; N is the number of units in the whole region; h=1, …, L represents the strata of explanatory variable *X*; $\sigma^{2}$ and $\sigma_{h}^{2}$ indicate the variance of *Y* in the whole region and in strata h., respectively; SSW represents the sum of squares. SST refers to the total sum of squares. A positive correlation is observed between the q value and the explanatory power of X on *Y*. A q value of 0 indicates that event *Y* is completely out of the control of variable *X*. A q value of 1 means that variable X fully controls event *Y*. The strata number L ranges from 2 to 10, and the variable *X* should be categorical.

In the interaction detector module, the interaction between different influencing factors can be identified. The interaction detector evaluates whether the influence of a factor on Y is independent or whether the explanatory power of the dependent variable Y decreases or increases by the interaction between *X*1 and *X*2. The q values of different factors and their interactions were estimated and then compared. The evaluation results were categorized into five conditions as follows. If the condition of $q\left(X1\cap X2\right)<\mathrm{Min}\left(q\left(X1\right),\;q\left(X2\right)\right)$ is satisfied, then the factor interactions will be nonlinearly weakened. If the condition of $\mathrm{Min}\left(q\left(X1\right),\;q\left(X2\right)\right)<q\left(X1\cap X2\right)<\mathrm{Max}\left(q\left(X1\right),\;q\left(X2\right)\right)$ is satisfied, then the uni-factor will be nonlinearly weakened. The interaction of the bi-factor will present an enhancement when satisfying the condition of $q\left(X1\cap X2\right)>\mathrm{Max}\left(q\left(X1\right),\;q\left(X2\right)\right)$. If the condition of $q\left(X1\cap X2\right)=q\left(X1\right)\;+\;q\left(X2\right)$ is satisfied, then the two factors are not related. When satisfying the condition of $q\left(X1\cap X2\right)>q\left(X1\right)\;+\;q\left(X2\right)$, the factor interaction will be nonlinearly enhanced.

The soil erosion process is influenced by both natural and human factors. Therefore, the following 10 different factors were chosen to explore the driving force, including precipitation (X1), temperature (X2), DEM (X3), slope (X4), aspect (X5), land use (X6), vegetation coverage (X7), soil type (X8), GDP (X9), and population (X10). A total of 13,481 sampling points were selected by creating a 2 × 2 km fishnet. Land-use data and soil type data were stratified according to their categorical system. Through the natural breakpoint method, factors were stratified into eight grades in each category when they were numerical. After inputting ten stratified factors, the corresponding q values can be obtained by executing the geodetector tool.

## 3. Results

### 3.1. Temporal and Spatial Variations in Soil Erosion in the TGRA

**Figure 2:**
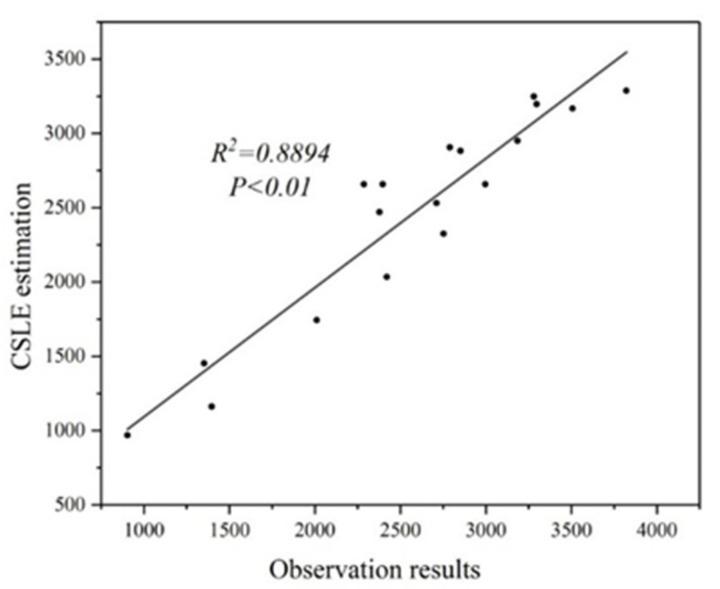
Comparison of soil erosion modules between the CSLE estimation and observation results from published papers on research in the TGRA.

After applying the CSLE model, the estimation result of the model was verified. Inspired by the meta-analysis approach, we searched for and collected fourteen published studies related to soil erosion estimation in the TGRA [53,58,59,60,61,62,63,64,65,66,67,68,69,70]. Then, the observed annual soil erosion module from the literature was selected to validate the estimation results of the CSLE model. Validation was performed by using 18 extracted pairs of data (Figure 2). The consistency of the calculation results and observation results proves the accuracy and applicability of the CSLE model in the TGRA.

**Figure 3:**
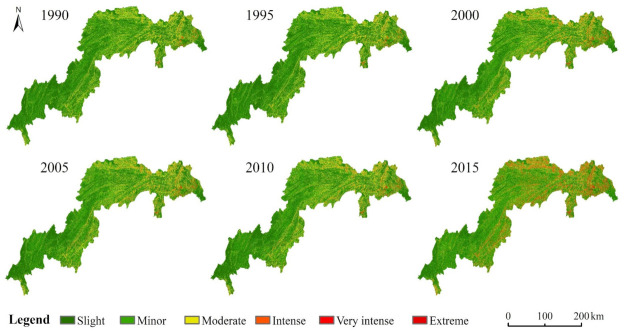
Spatial pattern of soil erosion degree in the TGRA from 1990 to 2015.

**Figure 4:**
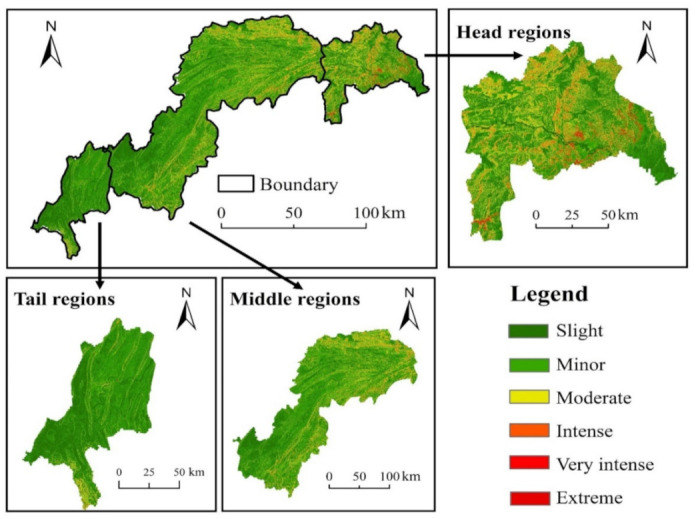
Spatial pattern of average soil erosion degree in the TGRA between 1990 and 2015.

Figure 3 shows the spatial pattern of soil erosion grades between 1990 and 2015. Figure 4 shows the spatial pattern of the average soil erosion degree, which is obtained by averaging the above six stages of soil erosion degree.

The soil erosion module in the TGRA has a moderate degree, reaching up to 2905.97$\;t\cdot {\mathrm{km}}^{-2}\cdot a^{-1}$, and its spatial pattern is generally consistent with the terrain in the TGRA, showing characteristics of being high in the east and low in the west. The most widely distributed soil erosion level in the TGRA is slight and minor, which has the largest area and occupies 79.55% of the total area. Very intense and extreme levels of soil erosion mainly occurred in areas with steep slopes and high elevations, such as Xingshan County, the northern part of Zigui County and Wushan County, accounting for 6.56% of the total area.

**Figure 5:**
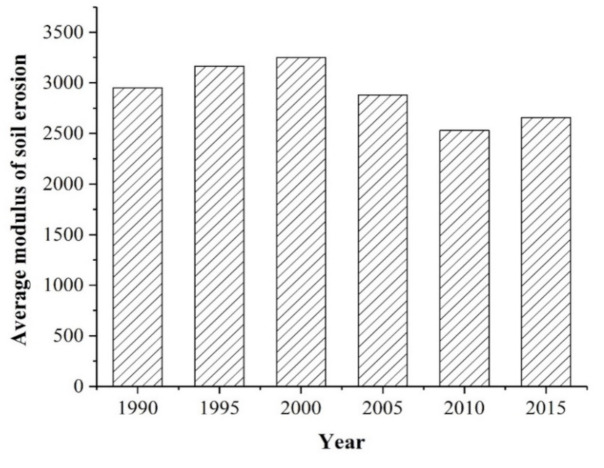
Average annual soil erosion module of the TGRA between 1990 and 2015.

Figure 5 exhibits an overall decline in the average annual soil erosion module between 1990 and 2015, showing an increasing and then decreasing trend. Over 25 years, the average annual soil erosion module decreased by 294.16 $t\cdot {\mathrm{hm}}^{-2}\cdot a^{-1}$, a decline of 9.97%.

The variable changes in the soil erosion area at all levels from 1990 to 2015 are shown in Table 2. The areas with slight and moderate levels of soil erosion decreased by 2.86% and 5.48%, respectively, in the total area. The area with minor soil erosion increased significantly, rising by 12.79% of the total area. The area with very intense and extreme levels of soil erosion has remained stable for 25 years. The overall soil erosion in the TGRA has generally been mitigated. In some areas of the TGRA, however, the soil erosion level has varied from low to high.

**Figure 6:**
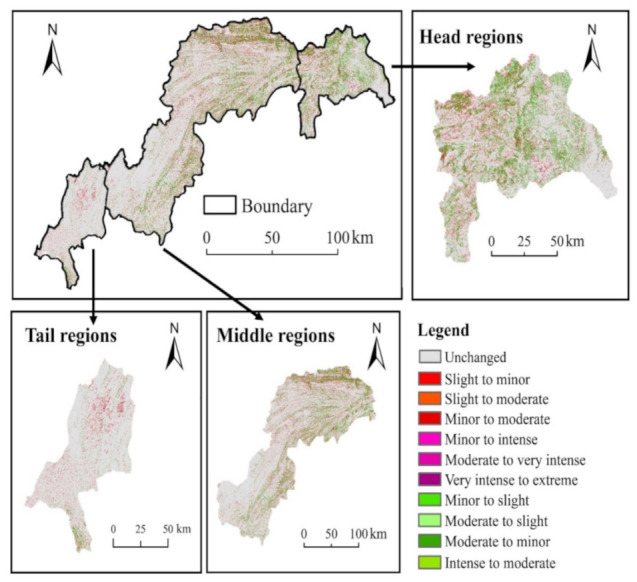
Spatial transition map of soil erosion grade from 1990 to 2015.

Figure 6 shows the spatial transition map of soil erosion at all levels from 1990 to 2015, and the area of the transition matrix is ranked statistically. The statistical results showed that within 25 years, no change had occurred in the soil erosion level of 74.82% of the total area. The three soil erosion levels with the most variation were the slight, minor and moderate levels. Approximately one-third of the area of slight soil erosion has transitioned to having more severe erosion. At the minor level, 18.57% of the soil erosion area has shifted to more severe erosion. At the moderate level, a total of 13.2% of the soil erosion area has shifted to more severe erosion, and 20.63% of that area has transitioned to a lower grade of erosion. The greatest variation in the transition of soil erosion levels was from slight to minor, from moderate to minor, and from moderate to slight.

The soil erosion areas that shifted from slight to minor levels were mainly distributed in areas with lower elevations, such as Yichang City, the areas surrounding downtown Chongqing, and areas along the Yangtze River, where the land-use types shifted from grassland to dry land and bare land. The soil erosion areas that shifted from moderate to minor levels were largely situated in Wuxi County, Xingshan County, and Zigui County, where the land-use types shifted from dry land to woodland.

### 3.2. Quantitative Attribution Analysis of the Spatial Heterogeneity of Soil Erosion

**Figure 7:**
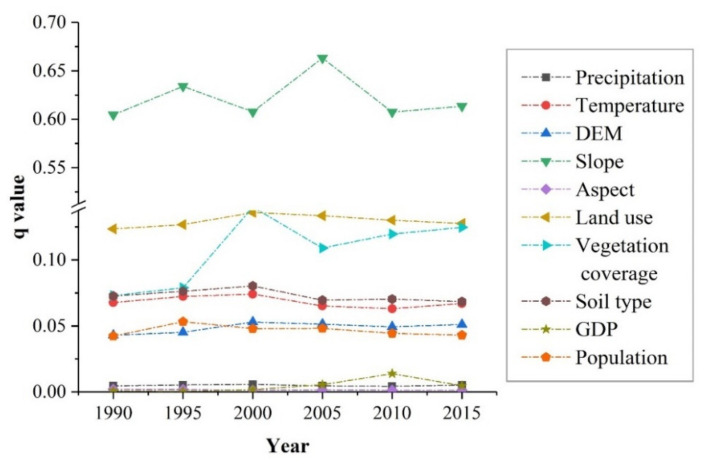
Influencing factors on soil erosion in the TGRA from 1990 to 2015.

Figure 7 shows the q values calculated for different influencing factors on soil erosion within the 25-year period. The results from the factor detector module showed that the explanatory power of influencing factors varied greatly (Table 3), and the q values from highest to lowest were slope (X4), land use (X6), vegetation coverage (X7), soil type (X8), temperature (X2), precipitation (X1), DEM (X3), population (X10), GDP (X9), and aspect (X5). Slope, land use, and vegetation coverage were the three dominant determinants over the 25-year period, and they differed significantly from the other factors, with average q values of 0.62, 0.13, and 0.11, respectively. Slope had the strongest explanatory power among the influencing factors. Soil erosion was less affected by population, GDP and aspect, and each had an explanatory power that was less than 0.1.

**Figure 8:**
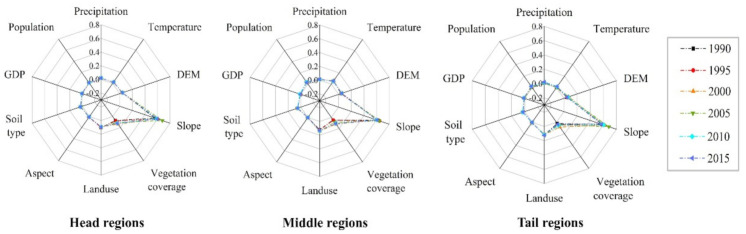
The  q values of influencing factors in different regions of the TGRA from 1990 to 2015.

Figure 8 shows the q value of influencing factors in different regions of the TGRA. The controlling factors affecting soil erosion were slope, land use and vegetation coverage. However, differences were observed in the factor sequence and explanatory powers.

In the head region of the TGRA, the three main factors in descending order were slope, vegetation coverage, and land use. Among the influencing factors, slope had the strongest explanatory power, with a q value of 0.602. The explanatory power of vegetation coverage achieved an average q value of 13.45% over the 25-year period, and this value was markedly higher than that in the other two regions. Land use had an explanatory power of 4.75%, which was far lower than that in the tail and middle regions.

In the middle region of the TGRA, the sequence of the factors was determined by slope, land use, and vegetation coverage. The slope still had the highest q value among the influencing factors, with an average explanatory power of 62.18% over the 25-year period. The explanatory power of land use reached an average q value of 13.29% over the 25-year period, which was notably larger than that in the head and tail regions. Vegetation coverage had an explanatory power of 8.73% over the 25-year period, which was slightly weaker than that in the head region of the TGRA.

In the tail region of the TGRA, the sequence of the factors was the same as that in the middle region. The slope was still the most explanatory influencing factor, with a q value of 0.611. The explanatory power of land use was slightly weaker than that in the middle regions, with an average q value of 11.67% over the 25-year period. The q value of vegetation coverage had an explanatory power of 4.59%, which was significantly smaller than that in the head and middle regions.

The results from the interaction detector module indicated that the interaction influence of any two factors was greater than that of a single factor to a large extent. The explanatory power of a single factor was obviously lower than that of interactions between factors. Table 4 shows the q values of three controlling interactions from 1990 to 2015. These interactions include the enhancements caused by land use and slope, vegetation coverage and slope, and land use and vegetation coverage. Moreover, the interaction q value remained relatively stable over the past 25 years.

**Figure 9:**
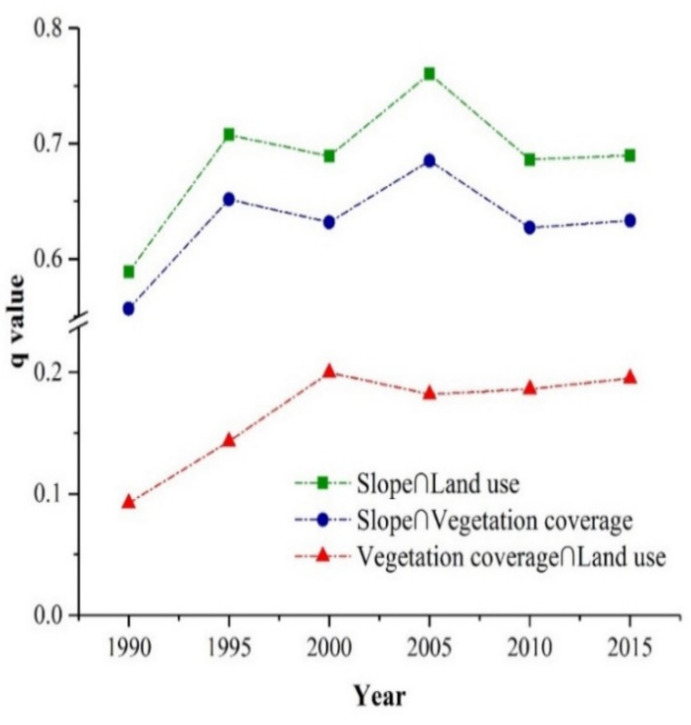
The three most dominant interaction factors in the TGRA between 1990 and 2015.

Figure 9 exhibits the three most dominant interaction factors between 1995 and 2015. Among them, the interaction between slope and land use was the most important controlling factor, with the highest q value of 70%. Soil erosion varied greatly under different slopes and similar land-use types and under various land-use types and similar slopes. For instance, the soil erosion in areas with a slope of 15° was considerably different from that of areas with a slope of 25° when the land-use type was cultivated land. When the slope was 25°, the soil erosion on cultivated land obviously differed from that on woodland. As the second controlling factor, the average q value of the interaction between slope and vegetation coverage was 0.64. The interaction between vegetation coverage and land use was the third controlling factor, with an average q value of 0.18. Additionally, the combinations of slope and other factors showed nonlinear enhancements. Adding the interaction with slope can greatly improve the interpretation ability of a single factor in relation to soil erosion.

**Figure 10:**
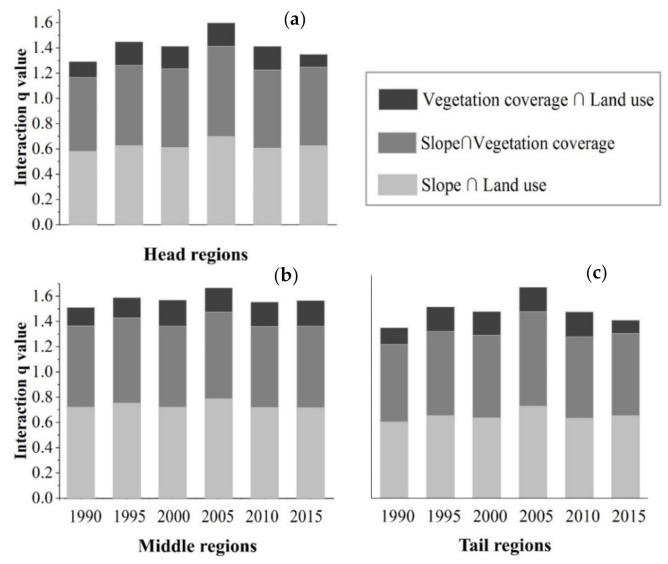
The three most dominant interaction factors in different regions of the TGRA from 1990 to 2015—(**a**) head region; (**b**) middle region; (**c**) tail region.

Figure 10 shows the discrepancies in the q values of the three most dominant interaction factors in the different regions. As the most controlling factor in all regions of the TGRA, the interaction between land use and slope in the head regions of the TGRA, however, was slightly lower than that between slope and vegetation coverage, with a q value of 58.3% in 1990. In the middle region of the TGRA, the explanatory powers of the three interaction factors were similar to those in the whole region of the TGRA. Over the past 20 years, the interaction between land use and slope had a stronger effect than that between slope and vegetation coverage, obtaining the greatest explanatory power of 76.7% in 2005. In the tail region of the TGRA, the q values of interaction factors were arranged in descending order as follows: interaction between slope and land use, interaction between slope and vegetation coverage, and interaction between vegetation coverage and land use. In 2005, the interaction between slope and land use had the highest explanatory power, with a q value of 0.83.

### 3.3. Soil Erosion Variation under the Influence of Slope and Land Use

Based on the results in Section 3.2, slope and land use were the two major determinants of soil erosion in the TGRA during the study period. Therefore, the effects of slope and land use on soil erosion variation were each investigated.

#### 3.3.1. Influence of Slope on the Spatial Heterogeneity of Soil Erosion

The spatial analysis was conducted by overlapping the soil erosion degree map and slope map. Statistical information for the area percentage of soil erosion at different slopes was collected (Table 5). Generally, the slope largely accelerated the occurrence of soil erosion, especially at soil erosion levels equal to or higher than moderate. Areas with slopes higher than 15° were more prone to soil erosion at moderate and above levels.

Soil erosion at different levels showed obvious spatial heterogeneity. Soil erosion at a slight level was mostly distributed in areas with slopes less than 25°, which accounted for 99.10% of the total area of slight soil erosion. Areas with slopes between 15° and 25° were more prone to a minor level of soil erosion, comprising 43.21% of the total area of minor soil erosion. Moderate soil erosion was generally scattered in regions with slopes larger than 25° and generally concentrated among slopes between 25° and 35°, accounting for 56.35% of the total area of moderate soil erosion. Areas with intense, very intense, and extreme soil erosion levels were mainly distributed in areas with slopes greater than 25°, and intense soil erosion usually existed in areas where the slope was greater than 35°, accounting for 83.25% of the total area of intense soil erosion. Very intense soil erosion mainly occurred in areas with slopes between 25° to 35°, accounting for 65.15% of the total area of very intense soil erosion. Areas with slopes greater than 35° were more likely to experience extreme soil erosion, affecting 68.92% of the total area of extreme soil erosion.

#### 3.3.2. Effect of Land Use on Soil Erosion Variation

The soil erosion process responds significantly to different land-use types. Table 6 exhibits the statistical information of the soil erosion modulus under different land-use types during six periods. Based on the corresponding soil erosion modulus, we sorted the land-use types. The descending order was as follows: bare land, dry land, paddy field, woodland, grassland, and construction land. The average annual soil erosion modulus of bare land achieved the largest value of 4022.42 t·km^−2^·a^−1^ in 2000. Chronologically, similar trends were detected for the soil erosion modulus under various land-use types, most of which represented an uptrend from 1990 to 2000, a downtrend from 2000 to 2010, and an increasing trend from 2010 to 2015. This result is consistent with the overall variation trend of the soil erosion modulus in the TGRA.

The soil erosion amount reflects the soil erosion status in terms of its quantity. We arranged the land-use type in descending order of area. The order was as follows: woodland, dry land, grassland, paddy field, construction land, and bare land. Among them, woodland comprised 47% of the total area, which was the largest area, followed by dry land. Bare land had the smallest area. Table 7 displays the calculation results for the soil erosion amounts under different land-use types. They are listed in descending order: woodland, dry land, paddy field, grassland, construction land, and bare land. Woodland had the largest soil erosion amount, accounting for 42% of the total amount of soil erosion in the TGRA. The amount of soil erosion in dry land was slightly lower than that in woodland, accounting for 37% of the total amount of soil erosion. Generally, differences in the soil erosion amount between woodland and dry land were not obvious.

Several conclusions can be formed by analyzing variations in soil erosion under different slopes and land uses. Areas with slopes greater than 15° were more subject to soil erosion. A severe level of soil erosion generally occurred in regions with slopes greater than 35°. Dry land had the most serious soil erosion among the land-use types. Areas of dry lands with slopes larger than 25° and steeply sloped farmland were crucial areas where soil erosion prevention and control can be targeted in the TGRA.

## 4. Discussion

This study offers an effective and feasible method to exploring soil erosion heterogeneity at temporal and spatial scales and investigating the driving forces in the TGRA by estimating the annual soil erosion modules using the CSLE model and investigating key determinants based on the geographical detector method. However, uncertainties may still exist, thus, the results must be interpreted with caution.

The soil erosion modulus in the TGRA calculated by the CSLE model was classified as moderate soil erosion overall, and its spatial distribution was consistent with the topographic trend, exhibiting an upward trend from west to east. The estimation results of the CSLE generally agreed with the results of similar studies in the TGRA [61,64,67,69], which demonstrates the applicability of the CSLE model in this study. However, some deviations still exist as a result of using different soil erosion estimation models. Every estimation model, such as the USLE, RUSLE, and CSLE, has specific factors. The calculation processes of each factor and data source are quite different. The above reasons lead to differences in the final soil erosion estimation results in the TGRA.

Soil erosion in the TGRA experienced three phases that corresponded to the three periods in the evolution of urbanization and national policy of China. Chronologically, soil erosion showed an overall downward trend, which was characterized by an initial increase, then decrease, and final increase. The TGD was approved and built in the first increasing phase of soil erosion from 1990 to 2000. The construction of the TGD has caused many ecological and environmental issues, such as immigration, relocation and land exploitation [71]. In some areas of the TGRA, soil erosion accelerated when the land-use type shifted from woodland and grassland to sloping farmland. In the decreasing phase from 2000 to 2010, the TGD was completed. The reservoir began to store water and generate electricity in 2006. With the implementation of the fourth phase of the immigration project from 2007 to 2009, the water level was stored at 175 m in the TGRA. The low-altitude area was submerged due to the high water level, resulting in a reduction in the erosion area. Furthermore, the ecological civilization construction was first proposed in the report of the 17th National Congress of Communist Party of China in 2007. With the advancement of ecological civilization construction, people’s environmental protection consciousness is increasing. In 2010, the average soil erosion modulus of the TGRA reached its lowest level, with a value of 2530.67 t·km^−2^·a^−1^. In the third increasing phase, which was increasing, from 2010 to 2015, the urbanization process in the TGRA accelerated with obvious urban expansion. Human economic activities such as deforestation and reclamation accelerated soil erosion and ultimately led to a growing trend in the soil erosion modulus during this period.

Generally, land-use variations have profound influences on soil erosion and should not be neglected. The degree of soil erosion transitioning from moderate to minor mainly occurred in areas of Zigui County, Xingshan County, Wuxi County, and Fengjie County, with a corresponding transition of land-use patterns from dry land to woodland. In addition, the shift in the soil erosion level from slight to minor was mainly distributed in areas with lower elevations, such as in Yichang City, areas around Chongqing City and areas along the Yangtze River, with the corresponding transition of the land-use pattern being from grassland to construction land. Consequently, the building of the TGD and urban expansion can provide explanations for the soil erosion variations.

In addition to land-use variations, the implementation of policies and programs also plays an important role in soil erosion control. Since 1989, the Chinese government has vigorously promoted a series of policies to protect the ecological environment over the last three decades, including the Soil and Water Conservation Program in the Upper Reaches of the Yangtze River (SWCP) [72], the Shelterbelt Program in the Yangtze River Basin (SPYRB) [73], the Transforming Sloping Cropland to Terraced Land (TSCTL) [74], the Grain for Green Program (GGP) [75], Natural Forest Protection Program (NFPP) [76], etc. The Chinese government has made great efforts in terms of soil erosion control by promulgating a series of ecological programs, and these efforts have achieved remarkable results [77,78]. The implementation of these policies can explain the variation in soil erosion during this period. However, the improvement of soil erosion is affected by multiple policies, which increases the difficulty of discriminating their effects. An interesting issue we hope to address in the future is to quantitatively determine the effect of a single policy or interactions of multiple policies on soil erosion improvement.

A large amount of sloped farmland is widely distributed in the TGRA, and our statistical analysis indicated that the average slope gradient is approximately 24°. According to the Chinese Soil and Water Conservation Act, the 25° slope corresponded to the maximum slope for cultivated land [42,79]. Thus, sloped farmland in the TGRA, especially in areas with slopes greater than 25°, must be monitored and governed. However, returning all sloped farmland with slopes greater than 25° to woodland is difficult. Moreover, during our field investigation, we found that the phenomenon of spontaneous abandonment of steeply sloped farmland by local farmers is very common in the TGRA. Since 1992, local farmers have changed their cultivation patterns on sloped farmland based on economic benefits, with a shift from traditional food crop farming to high-efficiency economic forest planting, such as fruit trees and tea trees [80]. According to the source-sink landscape theory, economic forests are a type of soil erosion sink and a source of non-point source (NPS) pollution. Cultivated land is a source of both soil erosion and nonpoint source pollution. Hence, for sloped farmland, functional conversion from cultivated land to economic forests will lead to a reduction in soil erosion and an increase in NPS pollution. Furthermore, filed survey showed that the amount of fertilization in orchard land is almost twice that of cultivated land. Consequently, the functional shift of sloped farmland from crop cultivation to economic forest planting would aggravate NPS pollution and become another challenge for public health [81,82], thus, it is obviously not conducive to the regional ecological sustainable development.

## 5. Conclusions

This study produced a feasible way to estimate soil erosion and quantitatively analyze its attribution in the TGRA over the past 25 years based on the CSLE model and the geographical detector method. Applying the above methodologies, several conclusions were drawn. The soil erosion in the TGRA generally improved from 1990 to 2015. Slope, land use and vegetation coverage were the three key determinants of soil erosion heterogeneity in the TGRA. The combinations of slope and other factors can play reinforcing roles in soil erosion occurrence. Slope and land-use types should be considered for the effective prevention of soil erosion. Sloped farmland, especially that with slopes higher than 25°, should be prohibited in the TGRA.

## Figures and Tables

**Table 1:** Grading standard of soil erosion in the TGRA.

Degree of Soil Erosion	Annual Soil Erosion Module $\left(t\cdot hm^{-2}\cdot a^{-1}\right)$
Slight	<500
Minor	500~2500
Moderate	2500~5000
Intense	5000~8000
Very intense	8000~15,000
Extreme	>15,000

**Table 2:** Percentage of soil erosion area at all levels in the TGRA from 1990 to 2015 (%).

Year	Slight	Minor	Moderate	Intense	Very Intense	Extreme
1990	33.69	44.41	16.90	2.88	2.08	0.03
1995	31.74	46.89	11.25	4.18	5.90	0.04
2000	31.16	45.79	5.21	10.53	7.27	0.03
2005	33.41	47.98	12.88	4.89	0.80	0.04
2010	36.60	46.81	10.34	5.31	0.91	0.04
2015	30.83	47.20	11.42	8.12	2.40	0.04

**Table 3:** Average q values of influencing factors in the TGRA from 1990 to 2015.

Influencing Factor	q Value
Slope	0.622
Land use	0.130
Vegetation coverage	0.107
Soil type	0.072
Temperature	0.068
Precipitation	0.005
DEM	0.049
Population	0.046
GDP	0.005
Aspect	0.002

**Table 4:** The q values of the three dominant interaction factors in the TGRA.

Year	Slope ∩ Land Use	Slope ∩ Vegetation Coverage	Vegetation Coverage ∩ Land Use
1990	0.672	0.615	0.151
1995	0.708	0.652	0.162
2000	0.689	0.632	0.200
2005	0.760	0.685	0.182
2010	0.686	0.627	0.186
2015	0.690	0.633	0.195
Average	0.701	0.641	0.179

**Table 5:** Statistical information for the area percentage of soil erosion at different slopes (%).

Soil Erosion Level	0°–8°	8°–15°	15°–25°	25°–35	>35
Slight	44.07	29.03	26.00	0.68	0.22
Minor	3.90	28.63	43.21	19.73	4.53
Moderate	0.01	0.17	7.00	56.35	36.46
Intense	0.01	0.02	0.59	16.12	83.25
Very intense	0.03	1.01	0.68	65.15	33.13
Extreme	0	0.84	5.82	24.43	68.92

**Table 6:** Average annual soil erosion modulus under different land-use types ($t\cdot {\mathrm{hm}}^{-2}\cdot a^{-1}$).

Land-Use Type	1990	1995	2000	2005	2010	2015
Paddy field	1837.35	1920.6	2020.9	1805.22	1792.24	1832.8
Dry land	2545.46	2561.19	2814.8	1862.61	1994.79	2356.19
Woodland	1807.59	1977.18	2282.22	1658.22	1606.27	1818.12
Grassland	1666.84	1751.4	1946.81	1341.36	1255.54	1323.49
Construction land	235.83	254.07	254.07	219.96	229.24	231.08
Bare land	3203.41	3683.93	4022.42	2892.6	3020.17	3144.11

**Table 7:** Soil erosion amount of different land-use types from 1990 to 2015 (t).

Land-Use Type	1990	1995	2000	2005	2010	2015
Paddy field	117.38 × 10^5^	121.98 × 10^5^	127.63 × 10^5^	112.52 × 10^5^	110.18 × 10^5^	108.02 × 10^5^
Dry land	374.11 × 10^5^	375.84 × 10^5^	411.86 × 10^5^	269.01 × 10^5^	283.16 × 10^5^	329.41 × 10^5^
Woodland	442.11 × 10^5^	483.85 × 10^5^	557.20 × 10^5^	408.33 × 10^5^	396.56 × 10^5^	447.21 × 10^5^
Grassland	111.16 × 10^5^	116.53 × 10^5^	129.86 × 10^5^	87.90 × 10^5^	81.89 × 10^5^	86.06 × 10^5^
Construction land	0.82 × 10^5^	1.04 × 10^5^	1.33 × 10^5^	1.36 × 10^5^	1.85 × 10^5^	3.12 × 10^5^
Bare land	0.29 × 10^5^	0.32 × 10^5^	0.35 × 10^5^	0.18 × 10^5^	0.18 × 10^5^	0.19 × 10^5^
